# Sphingobacterium Spritivorum Associated With Spontaneous Bacterial Peritonitis in a Cirrhotic Patient With Gram-Positive Bacteremia

**DOI:** 10.7759/cureus.26053

**Published:** 2022-06-17

**Authors:** Gift Echefu, Rameela Mahat, Silpita Katragadda, Karthik Reddy

**Affiliations:** 1 Internal Medicine, Baton Rouge General Medical Center, Baton Rouge, USA; 2 Internal Medicine Residency Program, Baton Rouge General Medical Center, Baton Rouge, USA; 3 Hospital Medicine, Baton Rouge General Medical Center, Baton Rouge, USA

**Keywords:** gram positive bacteremia, spontaneous bacterial peritonitis, immunocompromised patient, bacteremia, sphingobacterium spiritivorum

## Abstract

*Sphingobacterium spritivorum *(SS) is a ubiquitous gram-negative organism and an uncommon cause of infection in humans. To our knowledge, there are no reported cases of this bacterium causing spontaneous bacterial peritonitis (SBP) in patients with cirrhosis. In this report, we discuss a case of a male patient in his late 60s who presented with severe sepsis from methicillin-resistant *staphylococcus aureus *(MRSA)*,* in whom SS was subsequently identified via ascitic fluid culture. This unusual organism is known to have an innate resistance to multiple antibiotics and can cause life-threatening sepsis in cases of delayed or missed diagnosis. Clinicians should not be weighed down by anchoring bias and look for alternative, uncommon gram-negative organisms in cases of progressive sepsis in patients with ascites.

## Introduction

*Sphingobacterium spritivorum* (SS) is a glucose-nonfermenting, gram-negative bacillus that can be found in nature [1]. Very few cases of human infections associated with SS have been reported in the literature so far [2]. The elderly and immunocompromised are at risk of fatal diseases and the disease course can be complicated by the potential for antibiotic resistance [3-5]. Spontaneous bacterial peritonitis (SBP) is one of the common complications of end-stage liver disease (ESLD) with high morbidity and mortality rates. It complicates about 15-20% of all cases of decompensated ESLD with the associated mortality rate reaching about 10-46% [6,7,8]. Its mechanism involves the inadvertent translocation of bacteria from the bowel to the peritoneal cavity in the absence of non-surgical etiologies [9]. Portal hypertension with ascites, especially in cirrhotic patients, is an important predisposing factor [10]. Enteric gram-negative rods are the usual culprits in about 90% of cases with the most common being *Escherichia coli (E. coli)* and *Klebsiella pneumoniae* [11,12]. *Sphingobacterium* species are exceedingly uncommon causative agents. To our knowledge, no case of SS causing peritonitis in cirrhotic patients has been reported. We report the case of a male patient in his late 60s with alcoholic liver cirrhosis who presented with ascites and fever and in whom SS was eventually isolated from ascitic fluid.

## Case presentation

An elderly man in his late 60s presented to the emergency room for the management of hyperkalemia noted during workup at his doctor’s office. Further workup at the emergency room revealed elevated white blood cell count, predominantly neutrophilia. Blood cultures were obtained. He appeared clinically stable with no history or clinical indications of ongoing infection. His leucocytosis was then attributed to his taking oral steroids for alcoholic hepatitis that had been diagnosed in the past three weeks, and hence he was discharged from the emergency room to follow up with his primary care provider. Six days later, he presented to the hospital with generalized weakness and light-headedness after a witnessed fall at home. He had no fever, chills, abdominal pain, nausea, vomiting, diarrhea, confusion, or syncope. At this presentation, a review of blood culture reports received days earlier revealed methicillin-resistant *Staphylococcus aureus* (MRSA) and he was admitted for inpatient treatment with intravenous antibiotics with vancomycin and the management of decompensated liver cirrhosis. His past medical history was significant for alcoholic liver cirrhosis with grade I oesophageal varices, atrial fibrillation, hypothyroidism, essential hypertension, and polycythemia rubra vera. His home medications included levothyroxine, furosemide, spironolactone, nadolol, methylprednisolone, and folic acid.

On physical examination, his vitals were within normal limits except for hypotension (96/60 mmHg). He was awake and oriented but appeared lethargic, with scleral icterus, dry mucous membranes, and flapping tremors. Lung auscultation revealed diffuse coarse breath sounds, a heart rhythm that was irregularly irregular, and crackles over bilateral lung bases. The abdomen was distended, mildly tender, and firm with fluid wave. Skin exam revealed draining carbuncle on the posterior scalp, impetigo over the bridge of the nose, and numerous bruises and excoriations with scabs over the right lower abdomen, right hip, gluteal cleft, and buttock. Extremities were significant for pitting edema to the knees.

The patient's blood was taken for analysis including blood cultures. The laboratory data revealed hemoglobin of 17.9 g/dl and white blood cell count of 19.06 g/dL; the platelet count was 56 K/uL, international normalized ratio 1.8 IU, creatinine 2.01 mg/dl, sodium 125 mEq/l, potassium 6.5 mEq/l, aspartate aminotransferase 81 U/L (<34), alanine transaminase 95 U/L (<56), total bilirubin 10.1 mg/dL, lactate dehydrogenase 806 U/L (208-378), and albumin of 2.0 g/dl (3.2-4.8). Haptoglobin was within normal limits. Viral hepatitis panel was non-reactive for hepatitis A, B, C, and E. Blood culture repeated at presentation for admission, prior to antibiotics initiation, yielded MRSA. Abdominal CT was also obtained at presentation, which showed a small nodular-appearing liver compatible with cirrhosis with a moderate volume of ascites throughout the abdomen and pelvis. CT of the chest revealed patchy ground-glass opacities in the bilateral lower lobes as well as the right middle lobe, raising concerns for aspiration. Transoesophageal echocardiogram showed no evidence of vegetation or intracardiac or valvular pathologies. Paracentesis was performed under sterile conditions within 24 hours of admission, draining about 3 liters of ascitic fluid, which was assessed for biochemistry, cytology, and microbiology (Table 1). The ascitic fluid had a serum-ascites albumin gradient (SAAG) >1.1, indicating portal hypertension and a neutrophils count of 75.

**Table 1:** Ascitic fluid analysis WBC: white blood cells; SAAG: serum-ascites albumin gradient

Ascitic fluid	Results
Color	Pale yellow
Appearance	Cloudy
WBC	152 cells/uL (<200)
Neutrophilic count	49%
Lymphocytes	17%
Ascitic protein	0.6 g/dL
Ascitic albumin	0.4 g/dL
Serum protein	5.3 g/dL
Serum albumin	2.9 g/dL
SAAG	2.5 g/dL

For microbiology, the peritoneal fluid was incubated under anaerobic and aerobic conditions and then in MacConkey, blood, Columbia colistin, and nalidixic agar plates, as well as cooked meat medium for two to seven days. Non-hemolytic, light yellow, catalase, and oxidase-positive colonies were noted on blood and limited growth MacConkey agar plates. The only isolate was SS identified using the VITEK 2 Gram-Negative Identification card. No other colonies were present and there was no growth under anaerobic conditions. It was sensitive to amikacin, cefepime, cefotaxime, ceftazidime, ciprofloxacin, gentamicin, levofloxacin, meropenem, piperacillin/tazobactam, tetracycline, and trimethoprim/sulfamethoxazole but resistant to ceftriaxone, tobramycin, and aztreonam.

The patient was admitted for septic shock secondary to MRSA bacteremia with the source being the impetigo on his nose and scalp carbuncle. The steroid was discontinued due to sepsis, and he was managed with intravenous vancomycin and nasal mupirocin. Intravenous octreotide, midodrine, and albumin were initiated for hepatorenal syndrome. He did not receive gram-negative coverage as the source of infection was only thought to be MRSA. Despite this management, he developed multiorgan failure evident as hypotension refractory to intravenous crystalloids and colloids, hypoxemic respiratory failure, and atrial flutter with rapid ventricular response refractory to antiarrhythmic and rate-limiting agents. He requested comfort care and declined further interventions, opting for a "do not resuscitate" code status. On hospital day three, his condition progressively worsened, and he died of cardiopulmonary arrest.

## Discussion

*Sphingobacterium* species are aerobic, non-motile, catalase, urease, and oxidase-producing gram-negative rods that are non-fermentative. They grow as yellowish, slightly convex, smooth, opaque, and non-hemolytic colonies on blood and MacConkey agar [1,3,13,14]. *Sphingobacterium* species are so named due to a high sphingophospholipid concentration in their membranes. In 1983, Yabuuchi et al. [2] identified 15 species of the *Sphingobacterium* genus based on their biochemical and genetic analysis. Of these, the majority of clinically significant human infections are caused by SS and *Sphingobacterium multivorum* [15]. In our case, the ascitic fluid culture isolate was SS formerly known as Centers for Disease Control group IIk-3.1. It was first described by Holmes et al. [1] in 1982 and, like others of the same genus, it is found freely in the environment isolated from water, soil, and plant material. Its ability to produce acids from carbohydrates and alcohols distinguishes it from other *Sphingobacterium *species. The most common sources of SS isolation in human diseases are blood and urine [1,2]. So far in the literature, only a few cases of SS-associated human infections have been reported. These include respiratory tract infections in a cystic fibrosis patient [16], cellulitis [17,18], native valve infective endocarditis [19], and bacteremia [20,21,22], a majority of which affects elderly and immunocompromised patients [3,4,23] (Table 2). In our case, the patient was on prednisolone for alcoholic hepatitis with MRSA bacteremia, in the setting of decompensated liver disease, further compromising his immune system.

**Table 2:** SS human infections in literature SS: *Sphingobacterium spritivorum*; CRBSI: catheter-related bloodstream infection; SBP: spontaneous bacterial peritonitis; M: male; F: female; ESRD: end-stage renal disease; DM: diabetes mellitus; MSSA: methicillin-susceptible *Staphylococcus aureus*; CHF: congestive heart failure; COPD: chronic obstructive pulmonary disease; ESLD: end-stage liver disease; MRSA: methicillin-resistant *Staphylococcus aureus*

Case report	Diagnosis	Year reported	Age (years)/sex	Comorbidities	Outcome	Source of isolation	Management
Marinella et al. [15]	Cellulitis	2002	72/M	Parkinson's disease	Complete recovery	Blood	Cefazolin, followed by ampicillin/sulbactam
Gupta et al. [19]	CRBSI	2016	80/F	ESRD, DM	Complete recovery	Blood	Trimethoprim followed by meropenem and ciprofloxacin
Anthony et al. [4]	Cellulitis	2016	89/M	Parkinson’s disease	Complete recovery	Blood	Piperacillin/tazobactam followed by amoxicillin/clavulanate
Tronel et al. [18]	Cellulitis	2003	84/M	Refractory anemia	Complete recovery	Blood	Amoxicillin/clavulanate
Koh et al. [13]	CRBSI	2013	68/F	Acute myeloid anemia on chemo	Died	Blood	Cefepime and then ciprofloxacin
Abensur et al. [17]	Infective endocarditis	2019	61/M	Nephrotic syndrome, MSSA bacteremia	Died	Mitral valve, blood	Vancomycin, meropenem, and then oxacillin, changed to piperacillin/tazobactam
Hibi et al. [16]	Cellulitis	2017	80/M	CHF, COPD, tinea pedis	Complete recovery	Blood	Meropenem and then levofloxacin
Present case	SBP	2021	60s/M	ESLD, MRSA bacteremia	Died	Blood	Vancomycin, deceased prior to culture report

Of all the reported cases, cellulitis appears to be the most common manifestation (Table 2). This is likely due to its abundance in the environment and the skin being in contact with the elements. In our patient’s case, he had multiple traumatic skin lesions over the scalp, buttock, and abdominal area from minor injuries sustained while working in his garage. We think these may have been the entry sites for the bacteria. It is important to note that *Sphingobacterium* species in human infections are not usually considered contaminants; however, since it is abundant in the environment, when several cases are reported at the same institution, possible contamination should be considered [22,24]. This pathogen is rarely isolated in our laboratory and under the specific process explained in the case presentation section. Therefore, it is not considered a contaminant. Furthermore, cases of contamination are usually polymicrobial and often with *Staphylococcus* species isolation, which was not seen in this case.

Diagnosing SBP is based on positive ascitic fluid bacterial cultures and the presence of an increased ascitic fluid absolute polymorphonuclear neutrophil (PMN) count >250/mm^3^ in the absence of an obvious intra-abdominal surgically curable cause of infection. Noteworthy are other less frequently occurring variants of SBP that do not fit into the usual diagnostic criteria for SBP (Table 3). These include culture-negative neutrocytic ascites (CNNA), polymicrobial bacterascites, and monomicrobial non-neutrocytic bacterascites (MNNA) [25]. Consequently, there is a potential risk for delayed diagnosis, which has implications for management as these have similar rates of morbidity and mortality as SBP [26]. In our case, the patient had MNNA with the hallmark being the isolation of single colony organisms from the ascitic fluid culture with polymorphonuclear cell count <250 in the setting of clinical symptoms. The pathogenesis of this variant remains unclear. Several proposed explanations include ascitic fluid analysis done in the early phase of SBP in which neutrophilic response is still developing or one that has spontaneously resolved due to robust host defenses [25]. In some cases, bacterascites may be considered a contaminant especially if the culture is also positive for a common skin commensal bacteria or polymicrobial growth, in which case it should be considered a traumatic paracentesis [27]. MNNA without treatment may progress to SBP within hours and should be treated as promptly as SBP as they have similar mortality rates [26].

**Table 3:** SBP variants and ascitic fluid analysis SBP: spontaneous bacterial peritonitis; PMN: polymorphonuclear leukocytes

Variants of SBP	Ascitic fluid analysis		Comments
SBP (culture-positive)	PMNs ≥250 cells/mm^3^	Positive culture	Patients with cirrhosis and ascites, positive culture, with or without suggestive signs and symptoms
CNNA (culture-negative neutrocytic ascites; culture-negative SBP)	PMNs ≥250 cells/mm^3^	Negative culture	May indicate resolution of infection, poor culture technique, and prior antibiotics. Common phenotype; requires antibiotic therapy
Monomicrobial non-neutrocytic bacterascites (MNNA)	PMNs <250 cells/mm^3^	Positive culture	Ascitic fluid infection may resolve spontaneously or progress to SBP. Similar mortality to SBP and should be treated with antibiotics
Polymicrobial bacterascites	PMNs <250 cells/mm^3^	Positive culture	May indicate contamination, not true SBP

Bacteremia may coexist with SBP. Invasive gastrointestinal procedures such as catheterization or endoscopic operations, as well as gastrointestinal hemorrhage, may predispose individuals to bacteremia [28,29]. Unfortunately, it is associated with a poor prognosis, being an independent risk factor for short-term mortality in patients with SBP [30]. Gram-negative rods such as *E. coli* and *Klebsiella* are the most common isolates in ascitic fluid as well as blood cultures in patients with simultaneous bacteremia [30]. Gram-positive organisms are less common isolates and constitute about 25% of cases [11,12]. In certain situations, as in our patient, the ascitic fluid isolate may differ from the blood culture isolate, requiring broad-spectrum coverage.

Diseases caused by *Sphingobacterium *species appear to be virulent due to its variable antibiotic resistance to commonly used antibiotics, highlighting the need for effective coverage [1,3]. Several studies report susceptibility to β-lactams (penicillin derivatives, cephalosporins, carbapenems), fluoroquinolones, trimethoprim-sulfamethoxazole, and tetracyclines [14,16]. Remarkably, numerous strains of *Sphingobacterium* reported by Holmes et al. [1] and Yabuuchi et al. [3] as well as newer authors produce β-lactamase and are resistant to penicillin derivatives, cephalosporins, tetracyclines, aminoglycosides, aztreonam, and polymyxins [4,5,14,16,22].

Oftentimes, therapy for the MNNA variant of SBP is delayed by two to three days due to the time it takes for culture to become positive. In our case, the patient was appropriately managed with vancomycin for the prevailing MRSA bacteremia. He, unfortunately, succumbed to multisystem organ failure prior to the culture speciation with SS and sensitivity report.

## Conclusions

This case report of SS described an uncommon gram-negative organism as a potential cause of SBP in cirrhotic patients with ascites, further contributing to the studies on a number of uncommon but clinically significant infections caused by this organism. Immune compromise is an important risk factor and bacteremia portends a poor prognosis. Individualized therapy based on susceptibility data is critical considering the varied drug resistance and sensitivity patterns observed in diverse strains. This case emphasizes the importance of maintaining a high index of suspicion for SBP by gram-negative organisms in patients with ascites who present with gram-positive bacteremia and considering broad SBP coverage, particularly if there is progressive clinical decline despite adequate gram-positive coverage.
